# In Vitro and In Vivo Models to Assess the Immune-Related Effects of Nanomaterials

**DOI:** 10.3390/ijerph182211769

**Published:** 2021-11-10

**Authors:** Diana Boraschi, Dongjie Li, Yang Li, Paola Italiani

**Affiliations:** 1 Shenzhen Institute of Advanced Technology (SIAT), Chinese Academy of Sciences (CAS), Shenzhen 518055, China; lidj@siat.ac.cn (D.L.); yang.li@siat.ac.cn (Y.L.); 2 Institute of Biochemistry and Cell Biology (IBBC), Consiglio Nazionale delle Ricerche (CNR), 80131 Napoli, Italy; paola.italiani@ibbc.cnr.it; 3 Stazione Zoologica Anton Dohrn, 80121 Napoli, Italy

**Keywords:** cell lines, experimental models, immunosafety, immunotoxicity, in vitro models, in vivo models, nanomaterials, organoids, organs-on-chip, personalized testing

## Abstract

The immunological safety of drugs, nanomaterials and contaminants is a central point in the regulatory evaluation and safety monitoring of working and public places and of the environment. In fact, anomalies in immune responses may cause diseases and hamper the physical and functional integrity of living organisms, from plants to human beings. In the case of nanomaterials, many experimental models are used for assessing their immunosafety, some of which have been adopted by regulatory bodies. All of them, however, suffer from shortcomings and approximations, and may be inaccurate in representing real-life responses, thereby leading to incomplete, incorrect or even misleading predictions. Here, we review the advantages and disadvantages of current nanoimmunosafety models, comparing in vivo vs. in vitro models and examining the use of animal vs. human cells, primary vs. transformed cells, complex multicellular and 3D models, organoids and organs-on-chip, in view of implementing a reliable and personalized nanoimmunosafety testing. The general conclusion is that the choice of testing models is key for obtaining reliable predictive information, and therefore special attention should be devoted to selecting the most relevant and realistic suite of models in order to generate relevant information that can allow for safer-by-design nanotechnological developments.

## 1. Introduction: Why Nanoimmunosafety Is Important

### 1.1. Nanotechnology and the Use of Engineered Nanoparticles

Nanotechnology is one of the major technological advancements of the 21st century, with applications in many fields and products. Engineered nanoparticles (ENPs; i.e., particles with dimensions of 1–00 nm) can be produced with different materials as single particles or composites, can be designed in different sizes and shapes and can be surface-modified with different functional groups/molecules. The most produced ENPs worldwide are silicon dioxide (SiO_2_), titanium dioxide (TiO_2_) and zinc oxide (ZnO) ENPs [1], with applications in electronics (SiO_2_), manufacturing and construction (e.g., car tires, concrete, sports equipment, SiO_2_, TiO_2_, but also aluminum oxide, Al_2_O_3_ and carbon nanotubes or graphene), food additives (SiO_2_, TiO_2_), food packaging and textiles (silver, Ag), paintings (TiO_2_), sunscreens and cosmetics (TiO_2_, ZnO) and many others [2,3,4]. From a biomedical point of view, many ENP formulations are in use for diagnostic and therapeutic scopes. In imaging applications, ENPs (in particular gold—Au—and iron oxide—Fe_x_O_y_—NPs) are used as contrast agents in a wide array of techniques, from fluorescence imaging to positron emission tomography [5,6,7,8]. In therapy, Fe_x_O_y_-NPs are used for treating anemia and iron replacement therapy and magnetic fluid hyperthermia in tumours [9,10,11,12], while biodegradable polymeric or organic ENPs are already approved as drug delivery agents by the USA Food and Drug Administration and the European Medicines Agency, and are being currently developed for wider applications in cancer therapy, immunotherapeutic approaches and vaccination [13,14,15,16,17,18,19].

Because of their broad applications and abundant presence in our environments, it became urgent to implement a thorough evaluation of the possible toxic effects of ENPs on human and environmental health in order to identify and manage the associated risks [20,21]. As a consequence, many “safe-by-design” strategies have been developed during the past decades in the attempt to design safer nanomaterials [22].

### 1.2. Immunological Safety: How the Immune System Works

Within the significant progress in our knowledge of the interaction between engineered nanomaterials (ENMs0 and living organisms, evidence has surfaced that some ENPs can affect immunity by inducing cell stress and/or toxicity, or cause unwanted immune reactions [23,24].

The need for testing the immunological safety of any agent we knowingly or deliberately come into contact with or introduce in the environment stems from the awareness that immunity is the major defensive system that protects living organisms, and that any damage caused to the immune system may lead to significant harm, disease and death. Immunosafety does not exclusively pertain to human beings, but it should be sought for all living organisms, as all of them display immune defences based on impressively similar mechanisms [25,26]. Immunity can be broadly divided into two branches, innate and adaptive immunity. Innate immunity encompasses a number of different defensive mechanisms constitutively displayed by professional (e.g., phagocytes) and non-professional (e.g., epithelial cells) innate cells. Innate defensive responses aim at eliminating potentially dangerous external agents, such as infectious microorganisms, particles and toxins, but are also active towards endogenous threats, such as senescent or dying/dead cells or anomalous/transformed cells. The vast majority of living organisms, from plants to cartilaginous fish, only displays innate immunity. Innate immune cells can detect, kill or engulf and degrade foreign/anomalous entities, while innate factors, such as complement, can travel the organism to detect and attack foreign/anomalous objects. Innate reactions begin upon recognition of the possible danger, and are, in a way, automatic, i.e., they include the same array of reaction tools/events (e.g., production of reactive oxygen species (ROS), phagocytosis and intracellular degradation, degranulation and release of pre-formed mediators), their overall difference being based on the types and number of involved/reacting cells and factors and the microenvironmental conditions of the affected tissue (e.g., oxygen levels, temperature, presence of other cells, hormones, neurotransmitters and other immune/inflammatory factors) [24,25,26,27]. Adaptive immunity is a much more recent evolutionary development of immune defences, and is present in jawed vertebrates, from bony fish up to human beings, in parallel with innate immunity (which is present throughout the entire evolutionary scale). Adaptive immunity is based on the recombination of genes upon contact with a stimulus that gives rise to a number of new molecules (antibodies, T cell receptors), shaped so as to specifically recognize the foreign agent and mark it for elimination [25]. Thus, adaptive immunity complements innate immunity in higher vertebrates to afford an increased specificity of recognition and a more targeted elimination. Most likely, adaptive immunity has developed in order to achieve more specific defences, and thus be less prone to causing collateral damage to the body tissues in organisms that are physically complex (in which the damage to one organ may compromise the entire organism) and are widely mobile (thus exposed to a variety of new infectious or dangerous challenges by moving between different environments). The drawback of adaptive immunity is its reaction time that, because of the need for developing new specific defensive tools, takes several days to weeks before being fully effective. Innate immunity, on the other hand, is fast, and it can protect the body in its non-specific way, allowing for sufficient time to adaptive immunity to develop [24,25,26,27].

### 1.3. Immunological Safety of ENPs: Need of Relevant Experimental Models

In this scenario, immunosafety related to ENMs differs from the common toxicity testing approaches because of the physical–chemical characteristics of ENPs, which resemble microbial agents in many aspects (size, ordered surface structure) but which are very different from other points of view (inability to multiply, passive cell entry mechanisms, different intracellular trafficking and degradation). Thus, the immune system tackles ENPs with the same mechanisms and tools used for microorganisms, but it may end up up-regulating a different set of reactions with unforeseen side effects [24,26,27,28,29,30,31]. As a consequence, nanoimmunosafety assays need to capture a number of important pieces of information in order to accurately predict real-life events, and should focus on the experimental models that better describe the immune reactions to particulate agents, i.e., those used for eliminating microorganisms, apoptotic bodies and cellular debris. Of the many experimental immunosafety models available, none can fully predict such events, although they can provide some useful and informative results. The nanotoxicologist should therefore have the goals of the assessment clear in mind, so as to be able to select the most suitable experimental model or set of models. In order to be able to perform such selection, we should be aware of the pros and cons of the different models.

## 2. Immune Recognition and Response: Distinguishing between the “Normal” Defensive Response to Nanomaterials and Its Pathological/Damaging Dysregulation

**Figure 1:**
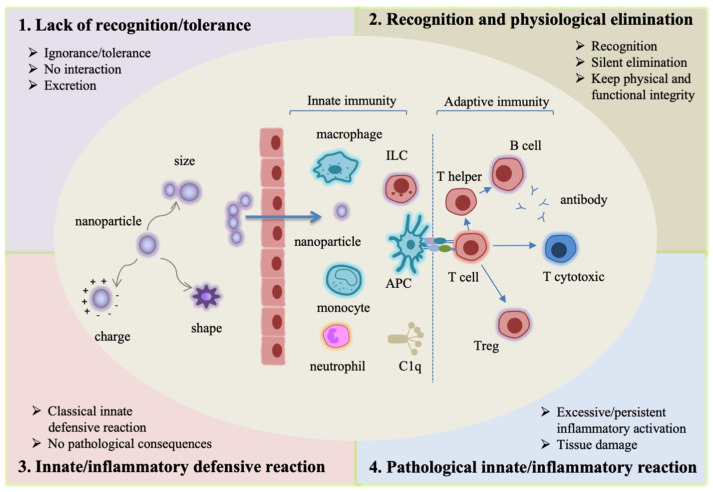
**The four possible outcomes ofnanomaterial–immune system interaction.** The reaction of innate immune cells to ENMs depends on the physical–chemical characteristics of the material and on the tissue microenvironmental conditions in which it occurs. Innate immunity is the first system involved in the interaction and is later responsible for initiating adaptive immune responses. The four possible outcomes are (**1**) Ignorance/tolerance, (**2**) Recognition and silent elimination, (**3**) Innate defensive reaction, (**4**) Pathological reaction. The latter event is very rare.

It is important to know that the interaction of nanomaterials with the immune system largely occurs with innate immune cells and effector molecules. This is always the case for plants and invertebrates, but it is also the case in organisms that display both innate and adaptive immunity. In fact, innate cells and soluble effectors are abundantly located in barrier tissues (skin and mucosal surfaces in vertebrates), are residing in all tissues for their scavenging/cleaning role and are also abundant in circulation, with the scope of replenishing the tissue pools and intervening in the case of peripheral damage or infection [25,32]. Broadly, the encounter of nanomaterials with the innate immune sentinels can lead to four types of outcomes (Figure 1).

1. Lack of recognition/tolerance. This case encompasses two different events: ignorance (the immune system does not perceive the nanomaterial) and tolerance (the nanomaterial is detected by the immune system but not considered as a danger, and therefore does not trigger any reaction). Many ENMs fall into these two categories, and are eliminated as such through renal filtration and excretion with the urine and faeces [33,34,35,36]. Both particles <6 nm [33] and larger particles [34,36] can be excreted by renal filtration without causing a reaction. This kind of non-interaction results in rapid elimination without consequences for the organism.
2. Recognition and physiological elimination. As for many particulate matters, the immune system can recognize an ENM as a potential danger and start an elimination process. Elimination mostly occurs by the action of the mononuclear phagocyte system (MPS), which encompasses phagocytes, such as macrophages, which have the specific role of engulfing particles and fragments of damaged tissue (dying cells, misfolded proteins) and degrading them into phagolysosomes, with the final goal of maintaining the tissue’s physical and functional integrity. This mechanism of silent/physiological elimination occurs constantly in all tissues. This process is physiological and causes no consequences to the body [33,37,38,39].
3. Innate/inflammatory defensive reaction. This is a classical innate defensive reaction, when the innate immune system perceives an ENM as a potential danger that needs a powerful reaction to be eliminated. Most exogenous agents, such as microorganisms, trigger this kind of reaction, which involves several types of innate cells and soluble mediators. The ordered structure of nanomaterial surfaces resembles that of microorganisms, thereby facilitating their recognition by innate molecules (such as the complement component C1q) and receptors (such as the Toll-like receptors, TLR, and the scavenger receptors) and the consequent activation of an inflammatory reaction. When the triggering agent is successfully eliminated, the reaction ends with a mechanism of resolution and subsequent repair. In fact, as already mentioned, an innate/inflammatory reaction is a powerful non-specific response that not only targets the dangerous agent, but that can also damage the surrounding tissue. Thus, once the triggering agent is eliminated, the same innate cells involved in the defensive reaction (such as macrophages) are functionally redirected into anti-inflammation and tissue repair [32]. An innate/inflammatory reaction is not a pathological reaction, because its scope is the elimination of the dangerous agent and the re-establishment of tissue integrity, although it can cause transient damage and the death of a number of cells (both immune and bystander cells) [25,40,41,42,43].

4. Pathological innate/inflammatory reaction. In rare cases, an innate/inflammatory reaction may reach excessive levels or fail to resolve, thereby causing severe or permanent damage to the organism. This is the case of some microorganisms that can survive within macrophages [44] and, in the case of ENMs, it can happen with indigestible or toxic materials or with high aspect ratio particles that cannot be engulfed by phagocytes. In these cases, a significant cell death takes place, and the reaction becomes persistent, with the formation of new non-functional “scarring” tissue (as in the case of fibromas and granulomas) and the consequent impairment of tissue functionality [45,46,47,48]. Another circumstance that can lead to pathological inflammation is a chronic exposure, which may result in a persistent immune challenge and inflammatory activation, with a risk of chronic inflammation and consequent persistent tissue functional damage [49,50,51].

Thus, at the cellular level we can identify different changes in the balance between innate/inflammatory and anti-inflammatory mechanisms, linked to the cellular activation status. Taking the homeostatic condition as benchmark (no inflammation, strong anti-inflammatory anti-oxidant mechanisms), the encounter with nanomaterials can result in no reaction (no/little change in the inflammation/anti-inflammation balance, point one above), in a silent elimination reaction (with activation of mechanisms that to do imply an overt inflammatory reaction but that may lead to autophagy and non-inflammatory apoptotic cell death; point two above), or in a full inflammatory reaction. The latter response can result in the necrotic inflammatory death of a large number of the involved cells, which is the rule in defensive innate/inflammatory reactions but which does not imply pathological consequences. Thus, extensive death of immune cells occurs during an inflammatory reaction (points three and four above) independently of whether inflammation is transient or persistent.

Notably, the size and shape of ENPs, in addition to their chemical composition and surface charge, is of particular importance in the interaction with the innate immune system. At the cellular level, different engulfment mechanisms are activated by phagocytes depending on the size of the particles, with small particles taken up by clathrin- or caveolae-dependent/independent endocytosis, whereas microparticles are phagocytosed or taken up my micropinocytosis [52]. Shape and rigidity of particles also influence their interaction with phagocytes and the final outcome, with high aspect ratio particles being more prone to induce cell death [39], mainly due to mechanical stress, and with rigid particles being readily endocytosed as opposed to soft particles [27]. In vivo, nanosized particles are in general more toxic than microsized particles of the same chemical nature, most likely due to the different kinetics and biodistribution and to the more efficient elimination of large particles by the MPS [53].

The difference between effects on immune cells, affected tissues and the whole body are summarised in Table 1, which underlines the fact that immune cell activation and death is not an indication of pathological effects.

On these bases, it becomes very important for the nanotoxicologist to discriminate between normal immune responses and health-threatening reactions in order to correctly define the immunosafety profile of the various nanomaterials. Also, depending on the use of nanomaterials (e.g., medical use vs. unintentional exposure), immune recognition and efficient elimination could be a benefit (in the case of unintentional exposure) or a drawback (in the case of medically-used nanomaterials, except those that should target immune cells for being effective).

**Figure 2:**
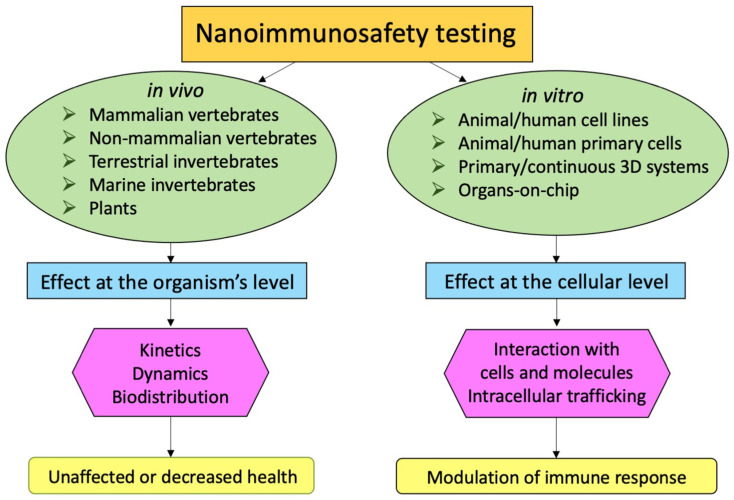
**In vivoand in vitro models for nanoimmunosafety testing.** The in vivo models used for immunosafety testing can provide useful information at the organism’s level, including biodistribution and elimination kinetics and possible pathological consequences. The in vitro models can unravel the cellular, subcellular and molecular mechanisms underlying the nano-immune interactions.

Thus, among the many experimental models available for testing the immunological effects of nanomaterials, it is important to select those that are more likely to provide realistic and predictive information. These can be different depending on the initial question. If the question is whether a nanomaterial can induce a chronic inflammatory pathology in human beings, examining the capacity of the same nanomaterial to activate innate immune cells in culture or to induce their death is inadequate, because inflammatory activation of innate cells mainly occurs without pathological consequences for tissues, organs or the entire organism. An evaluation of characteristics and applicability of the main available models is reported hereafter (summarized in Figure 2).

## 3. In Vivo Immunosafety Models, from Plants to Mammals

The value of in vivo models in nanoimmunosafety and immunocompatibility studies is undoubtable, and they are, in many instances, irreplaceable. At present, no other experimental system (in vitro, on chip, in silico) can provide information based on the complex network of biological interactions and cross-regulatory pathways that are triggered upon exposure to a nanomaterial (see below for the more recent in vitro organ-like models). In vivo models allow for a complete characterization of nanomaterial kinetics and biodistribution upon acute and chronic exposure, and are also useful for predicting effects in conditions of immunological frailty. These models are, however, not optimal for assessing the molecular interactions that occur between nanomaterials and immune cells, such as the intracellular trafficking and transformation, the interaction with biological molecules and the kinetics of their mutual effects at the cellular and subcellular level.

### 3.1. Selecting the In Vivo Nanoimmunosafety Model

The selection of the most suitable in vivo model depends on the goal, for instance, whether human or environmental immunosafety are concerned or whether the effects on innate or adaptive immunity are the focus. We all agree that humans are the best model for human health [54,55], as well expressed by Sydney Brenner: “We don’t have to look for a model organism anymore. Because we are the model organisms” [56]. However, when human data are not available, the most used in vivo models for predicting immune effects on human beings are mammalian organisms, in particular, mice. Immunosafety experiments on rats, rabbits and guinea pigs are increasingly abandoned, not only because of ethical issues, but also because of the limited availability of specific reagents (such as antibodies or recombinant proteins) and modified animals (with overexpression or deletion of specific genes). Studies on pigs and piglets are preferred for assessing complement activation [57], while for studies on vaccine efficacy against certain infections, ferrets, marmosets and other non-human primates represent the best choice [58,59,60]. Mouse models are by far the most widely used because of the ease of handling, the relatively low cost and the huge availability of reagents and tailored models. Genetically engineered, xenograft and humanized mouse models represent recent progress in terms of resembling human immune responses, and are a precious resource for immunosafety studies, as they allow us to assess the immunological effects of nanomaterials in a variety of disease conditions [60]. Naturalising laboratory mice is an additional way of increasing their similarity to human conditions, e.g., by changing the housing temperature, light–dark cycle, physical exercise and exposure to microbes to make their immune reactivity more similar to that exhibited by human beings [61]. Among non-mammalian vertebrates, several fish models are available, including the well-known zebrafish (*Danio rerio*), which displays both innate and adaptive immunity [62]. Zebrafish larvae are particularly useful for the study of innate immunity in vivo in vertebrates [63] and for assessing the immune effects of nano and micromaterials [64,65,66].

### 3.2. Drawbacks and Limitations of In Vivo Models

There are some major drawbacks that are limiting the use of in vivo vertebrate models. The first one is ethical, with health and regulatory authorities in Europe and the USA having controlled the use of experimental animals and encouraged the use of alternative methods (see the 3R policy, Replacement, Reduction, Refinement) [67]. The need for justifying and obtaining approval for each experiment on animals has the advantage of forcing scientists to select with great care their animal models as the most likely to obtain reliable results. The other important limitation is the fact that laboratory animals are not human beings, and although the general features may appear similar, there are many differences in immune responses and mechanisms. This is being explored in quite some detail due to the differences between mouse and man and the consequent possibility of translating the results obtained in the mouse to the human situation [59,68,69,70]. For instance, the inflammatory response can be very different between mouse and man and can use different mechanisms and pathways [70]. As an example, an important anti-inflammatory cytokine, IL-37 [71,72], and an important chemokine, IL-8 [73], do not exist in mice, which use different factors to obtain the same results in the control of inflammation. Also, different inbred mouse strains can have different immunological biases, as in the case of the Th1-biased response in C57BL/6 mice vs. the Th2 bias of BALB/c mice. Thus, nanotoxicologists should select their in vivo models with particular care to make sure that the selected model reliably represents the human response, relative to the specific parameter under examination, bearing in mind that different models may be needed for the realistic assessment of different parameters.

### 3.3. Common Immunological Features across Living Species

Several other experimental models are available which have been developed for studies of developmental biology and toxicology, and which are also useful for the assessment of nanoimmunosafety of environmental species. In addition, it is notable that several mechanisms of innate immunity are evolutionarily conserved, making several invertebrate models suitable for assessing innate immune reactions to nanomaterials in vivo in the absence of the confounding presence of adaptive immunity. Some immunological characteristics can be specific to the environment or living characteristics of the species (terrestrial vs. aquatic, bentonic/mobile vs. sessile/fixed), thus, it is more likely that an invertebrate human proxy would need to be terrestrial and mobile without a specific biological environment. Conversely, several basic mechanisms of innate recognition (through innate receptors and sensors) and engulfment/degradation by phagocytes are largely conserved and active with very similar mechanisms in every species. Thus, the model plant *Arabidopsis thaliana*, the woodlouse *Porcellio scaber*, the fruit fly *Drosophila melanogaster*, the nematode *Caenorhabditis elegans*, the earthworm *Eisenia fetida*, the blue mussel *Mytilus galloprovincialis*, the sea squirt *Ciona intestinalis*, the sea urchin *Paracentrotus lividus*, the cephalopod *Octopus vulgaris* and many others can be used for environmental nanoimmunosafety studies, but also as a proxy for human innate responses [26,30,74,75,76,77,78,79,80,81,82,83].

## 4. In Vitro Immunosafety Models: Cell Lines and Primary Cells

The use of in vitro models offers many practical advantages that make them widely used in immunotoxicological studies. These include high reproducibility, fast and easy experimental procedures and low costs. The type of information that can be obtained in vitro is also different from that provided by in vivo experiments, as in vitro systems allow for examining the direct interaction of nanomaterials with immune cells at the cellular, subcellular and molecular level in controlled conditions.

### 4.1. Transformed Cell Lines

Classical in vitro systems are based on continuous immortalized cell lines, i.e., tumour or otherwise transformed cells that have the advantage, compared to primary immune cells, of an indefinite number of cell divisions without undergoing senescence, which allows for an endless source of cells that display the same phenotypical and functional characteristics. An additional advantage of cell lines is that they can be genetically manipulated, with transient and stable transfection systems or other methods, in order to overexpress or silence some specific genes/functional characteristics. This would allow for a detailed analysis of interaction and activation pathways triggered by nanomaterials in specific immune cell types. Many human and mouse cell lines are available, usually derived from lymphomas or leukemias, representing all types of innate and adaptive immune cells (T and B lymphocytes, monocytes, macrophages, dendritic cells, eosinophils, basophils, neutrophils, NK, etc.). In invertebrates, a number of insect cell lines have been developed that can help assess in more detail the immune/defensive reactivity to external challenges [84]. A disadvantage of continuous cell lines is that their validity as a proxy of primary, normal cells is not obvious, and needs to be assessed for each cell line and each immune biomarker. Thus, for instance, when selecting a murine cell line for testing human nanoimmunosafety, this must be clearly justified as having unique advantages that make their use preferred to the use of corresponding human cell lines. This is particularly relevant because of the significant differences between mouse and man for some innate/inflammatory parameters (e.g., mouse immune cells are much less reactive to inflammatory stimuli than human cells) [85,86,87]. It should also be considered that cells from cell lines are usually at a differentiation stage that is not the same as in mature primary cells of the same lineage, with consequent differences in reactivity to stimuli and type and extent of response, in addition to the fact that immortalized cells continuously proliferate (and increase in number during culture) as opposed to primary cells. This is particularly important when testing nanoimmunotoxicity in terms of cell death or cell proliferation, as some nanomaterials may result as toxic on proliferating cells but totally safe on their primary non-proliferating counterparts. An example is the mouse cell line RAW 264.7, a peritoneal leukemic cell line with macrophage morphology. RAW264.7 shares some functions with primary macrophages, for instance phagocytosis, but substantially differs from primary cells in other aspects, such as spontaneous proliferation (which is high in RAW264.7 and absent in primary cells), tumoricidal activity and IL-1β production (significant in primary macrophages and absent in RAW264.7 cells) [88] (unpublished results). Thus, this cell line can be a good in vitro model for macrophage phagocytosis but not for macrophage cytocidal activation/M1 polarization or for testing cycle-dependent cell death.

### 4.2. Primary Cells

The use of short-term cultures of primary cells, isolated from blood or lymphoid/immune organs, has advantages and disadvantages as compared to immortalized cell lines. The main disadvantages are practical, and include the difficulty (in some cases impossibility) in sourcing and isolation, and the significant variability of response from donor to donor. This is less of a problem when using primary cells from inbred mice, and also in the case of human innate immunity, as in this case the donor-to-donor variability is limited, at least in qualitative terms [89]. Primary cells better reproduce the reactivity of normal cells in vivo and the variability of response observed in real-life conditions. However, assessing nanoimmunosafety on primary cells requires testing a significant number of donors in order to identify effects that can be attributed to the nanomaterial beyond the variability of response within the population. Although cumbersome, this kind of approach provides additional important information (which is lost with cell lines and inbred mice), i.e., that human beings and many other living species are different from each other and, based on their health/metabolic conditions and environmental context, they can mount different immune reactions to the same nano-challenge. This observation would suggest that a fully valid nanosafety assessment should be personalized, since immune reactivity depends on the individual conditions [90]. An important limitation of the use of primary immune cells, as mentioned above, is their sourcing. As immune cells are scattered through the organism and have different functional profiles depending on the organ in which they reside, primary cells of the same lineage but residing in different organs can have a completely different reaction to nanomaterial exposure [91]. Although this is known, the limitations in accessing immune cells have restricted immunosafety assessment to a few easily accessible cell types, e.g., blood leukocytes in humans, spleen and peritoneal cells in the mouse and haemocytes in several invertebrates, thereby missing the majority of immune reactivities at the organ level. While many tissue-resident cells can be dissected from animal organs, this is much less likely to occur for humans, being limited to some bioptic or autoptic tissues, a circumstance that makes immunotoxic assessment on human tissue-resident immune cells unfeasible. In the same context, it is important to note that even in cases of easier sourcing, as, for instance, mouse peritoneal macrophages, the macrophage population can considerably vary depending on the experimental protocol used for cell collection. Since the resident peritoneal cells are not abundant, many researchers inject some irritant (such as thioglycolate) intraperitoneally one to two days before cell harvesting, to increase the number of macrophages. However, the macrophage population collected after thioglycolate injection is phenotypically and functionally different from the resident cells, as it is composed of inflammatory cells mostly recruited from blood and participating in a local inflammatory reaction triggered by the irritant [92].

### 4.3. Good In Vitro Method Practices and Immunoactive Contaminations

Several issues should be considered when using in vitro assays of nanoimmunotoxicity. An exhaustive guidance on good in vitro method practices (GIVIMP) has been recently published by OECD [93] and includes all the major issues encountered in in vitro experimentation. Some of them, however, may need additional attention in the case of immunotoxicity, and are reported hereafter. A major case is that of contamination with bioactive agents. While immunologists are well aware of the problem, nanotoxicologists may need to pay attention to it. Contamination of continuous cell lines with mycoplasma is frequent (*Mycoplasma orale*, coming from the laboratory personnel, is the most common contamination), not eliminated by common antibiotics and can go undetected because cells may look perfectly healthy and unaffected. However, immune reactions of mycoplasma-contaminated cells can be significantly altered in comparison to uninfected cells, leading to the generation of false results [94]. Thus, cell lines should be regularly tested for mycoplasma contamination to ensure that experiments are run with mycoplasma-free cells. In the case of primary cells, mycoplasma contamination is not an issue [94]. Another contaminant that could lead to misinterpreting the toxicity results is the contamination with bacterial LPS. LPS is a potent inducer of inflammation, in human cells in particular, which is ubiquitous and resistant to common sterilization procedures. All cell culture labware and reagents are generally endotoxin-free, but the possible contamination of nanomaterials needs to be assessed to prevent attributing immune effects to them that are actually caused by contaminating LPS [95,96,97]. It should be noted that different nanoparticle batches produced in the same lab with the same procedure can have different endotoxin contamination, underlying the frequency of unwanted contamination and the need for accurate testing [96]. The same caution should be adopted for in vivo experiments, being aware that different animals and different cells/cell lines are sensitive to the activating and toxic effects of LPS in different ways (with human monocytes and dendritic cells being very sensitive, mice less sensitive and marine invertebrates insensitive).

## 5. Conclusions and Future Perspectives: Addressing Biological Complexity for a More Realistic Nanoimmunosafety Assessment

In vitro culture systems have significantly developed in the last several years to reproduce the in vivo situation in greater detail. Since immune cells are present in all organs and tissues, advanced immunosafety testing should be performed in systems that represent the microenvironmental conditions of different tissues. A system based on a single cell type (human monocytes) was designed to reproduce the kinetics of the tissue’s microenvironmental changes (temperature, oxygen tension, plasma influx, cytokines and growth factors) during a physiological/resolving inflammatory reaction [89], and used for assessing the possible impact on metal nanoparticles [98]. The use of 3D cultures on a collagen matrix, as opposed to the conventional 2D cultures on a plastic surface, also showed that human macrophages behave differently in response to metal nanoparticles [99], supporting the important notion that the innate immune cells’ capacity to sense changes in the surrounding space strongly influences their reactivity [100].

Many complex in vitro models have been designed which assemble different cell types in a tissue-like composition and structure. From the initial use of cell lines, to reproduce epithelial and immune cells in barrier tissues, such as the gut in transwell cultures [101,102], we can now exploit more complex models based on primary cells derived from human organoids or iPSC, assembled in a 3D organ-like architecture in microfluidic devices that reproduce the fluid movements occurring in vivo (a key advantage vs. static cultures in terms of likelihood of exposure and contact with nanomaterials). Such microfluidic and organ-on-chip systems are very promising for future regulatory adoption, because they join the advantages of being based on human non-transformed cells and reproducing the complex cell–cell interactions within an organ with the possibility of using controlled exposure protocols and high content detection systems for assessing the immune reaction of each individual cell within the system. These systems are, however, still at an early stage, as they still cannot realistically include many important players. Again, using the gut as an example of, the presence of commensal microbiota is essential [103], not only for attaining a realistic interaction with ingested nanomaterials [104], but also in determining the reaction of gut-associated immune cells [105].

Organ-specific immune responses are also powerfully controlled by neurotransmitters and hormones [106,107]. It should be noted that, when some nanomaterial arrives in the gut, it certainly will not arrive as a pristine material, but it will have interacted with a number of external (food components, allergens, other dusts and particles) and endogenous (saliva, bacteria, digestive enzymes, mucus) agents. Thus, future models should consider that the immune systems of humans and environmental species will face ENMs combined with other agents, either bystander or bioactive compounds, and that the immune response will be raised againts the combination. Eventually, the interindividual variability due to age, diseases, metabolism and microbiota composition lets us foresee the development of a precision nanoimmunosafety assessment, using in vitro advanced models designed based on individual characteristics.

A summary of the advantages and drawbacks of the currently available models for nanoimmunosafety assessment is reported in Table 2, and could serve as a guide in the selection of the most suitable models.

## Figures and Tables

**Table 1:** The innate immune reaction to ENMs at the level of cells, tissues/organs and the entire organism.

Innate Reaction	Cellular Level	Tissue/Organ Level	Organism Level
Ignorance/tolerance	no effect	no effect	no effect
Silent elimination	activation	no effect	no effect
	autophagy, apoptosis		
Resolving inflammation *Strong reaction with eventual elimination*	inflammatory death of immune and bystander cells	transient damage	no effect
Chronic inflammation *Strong non-resolving reaction*	continuous inflammatory death of immune and bystander cells	persistent damage, tissue destruction and neoformation	pathology

**Table 2:** Advantages and disadvantages of in vivo and in vitro immunosafety models.

Models	Pros	Cons
*in vivo*	Assessment within the complex network of biological interactions and cross-regulatory pathways Nanomaterial biodistribution and elimination kinetics can be examined Availability of genetically engineered, xenograft and humanized models Naturalised or wild animal models can capture the population’s complexity Non-mammalian models available for innate immunity In vivo high content imaging techniques can identify nano-cell interactions Realistic results	Difficulty in assessing the interaction between nanomaterials and immune cells and its biological consequences Difficulty in assessing the interaction between nanomaterials and biological molecules Limited availability of specific reagents for some animal models Ethical issues Differences in immune responses and mechanisms respect to humans
***in vitro***	*Cell lines* High reproducibility, fast and easy experimental procedures and low costs Direct interaction of nanomaterials with immune cells at the cellular, subcellular and molecular level in controlled conditions Endless source of cells with the same phenotypical and functional characteristics (no senescence) Genetic manipulation is possible *Primary cells* Reactivity of normal cells in vivo and variability of response observed in real-life conditions Direct interaction of nanomaterials with immune cells at the cellular, subcellular and molecular level in controlled conditions *Organs-on-chip* Possibility of using human non-transformed cells Cell–cell interactions in a tissue-mimicking context (other cell types, 3D arrangement, extracellular matrix, relevant fluidic and gaseous conditions, etc.) Possibility to reproduce disease conditions	*Cell lines* Different biological characteristics vs. primary cells (e.g., continuous proliferation, polyploidy) Different reactivity vs. primary cells Predictivity needs to be validated for each endpoint/biomarker Use of animal cell lines for human risk assessment needs validation *Primary cells* Difficult sourcing and isolation Limited availability (e.g., human tissue-resident immune cells) Limited survival in culture (senescence) Risk of anomalous reactivity of cells outside their tissue context (e.g., different extracellular matrix, contact with other cells, three-dimensional arrangement, oxygen tension, etc.) Variability of response from donor to donor *Organs-on-chip* High costs Complex set up and experimental running Difficulty in achieving a full in vivo-mimicking tissue complexity Difficulty in obtaining accurate identification of interactions and effects
